# Supplementary material for: Mitochondrial KMT9 methylates DLAT to control pyruvate dehydrogenase activity and prostate cancer growth
Source: Nat Commun. 2025 Jan 30;16:1191. doi: 10.1038/s41467-025-56492-8 (PMC11782658; doi:10.1038/s41467-025-56492-8)
Supplement: Supplementary file 1 — Supplementary Information [file 41467_2025_56492_MOESM1_ESM.pdf]

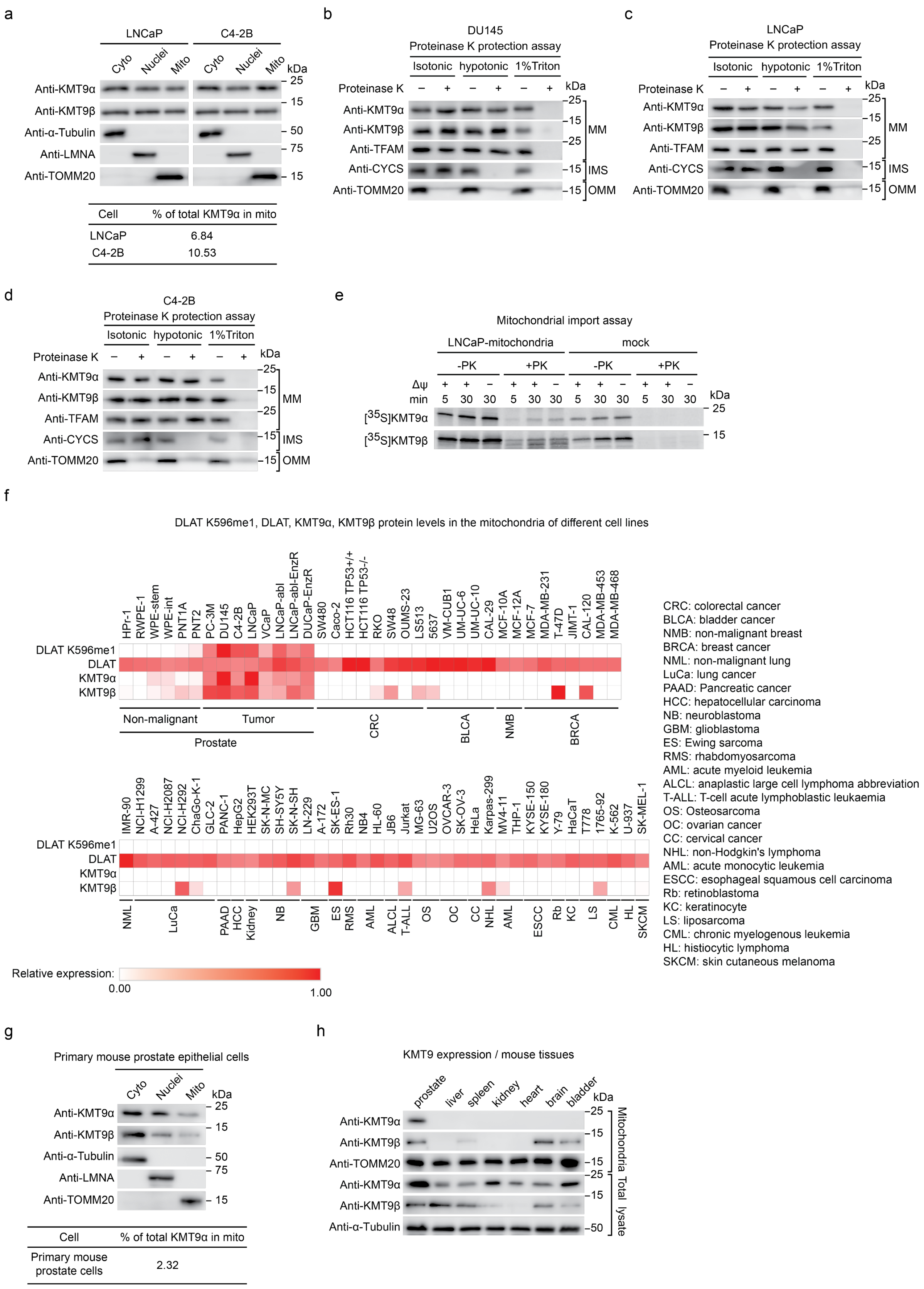

Supplementary Figure 1. KMT9 is localized in mitochondria of PCa cells. a, Analysis of cytosolic, nuclear, and mitochondrial fractions of LNCaP and C4-2B cells by Western blot

using anti-KMT9α and -KMT9β antibodies. Purity of cytosolic, nuclear, and mitochondrial fractions was verified with antibodies directed against α-Tubulin, LMNA, and TOMM20, respectively. The percentage of total KMT9α in mitochondria was quantified by densitometric analysis of Western blots. **b**-**d**, Proteinase K protection assay for mitochondria isolated from DU145 (**b**), LNCaP (**c**), or C4-2B (**d**) cells and assayed in isotonic, hypotonic, or 1% Triton X-100-containing buffer. Mitochondrial proteins TFAM (mitochondrial matrix, MM), CYCS (intermembrane space, IMS), and TOMM20 (outer mitochondrial membrane, OMM) were used to monitor the extent of digestion. Western blots were decorated with the indicated antibodies. **e**, Import of [^{35}S]-labeled, in vitro translated KMT9α and KMT9β into mitochondria isolated from LNCaP cells. Imported proteins were detected by autoradiography. Δψ, membrane potential; PK, proteinase K. **f**, Relative levels of DLAT K596me1, DLAT, KMT9α, and KMT9β in mitochondrial extracts of diverse cancer and non-cancer cell lines were revealed by Western blot analyses using the indicated antibodies. **g**, Presence of KMT9α and KMT9β in cytosolic, nuclear and mitochondrial fractions of primary mouse prostate epithelial cells analyzed by Western blot. **h**, Presence of KMT9α and KMT9β in mitochondrial extracts and total cellular lysate of mouse tissues was revealed by Western blot analysis using the indicated antibodies. LMNA, TOMM20 and α-Tubulin served as controls (**g**, **h**). **a**-**h**, All experiments were independently repeated at least three times with similar results.

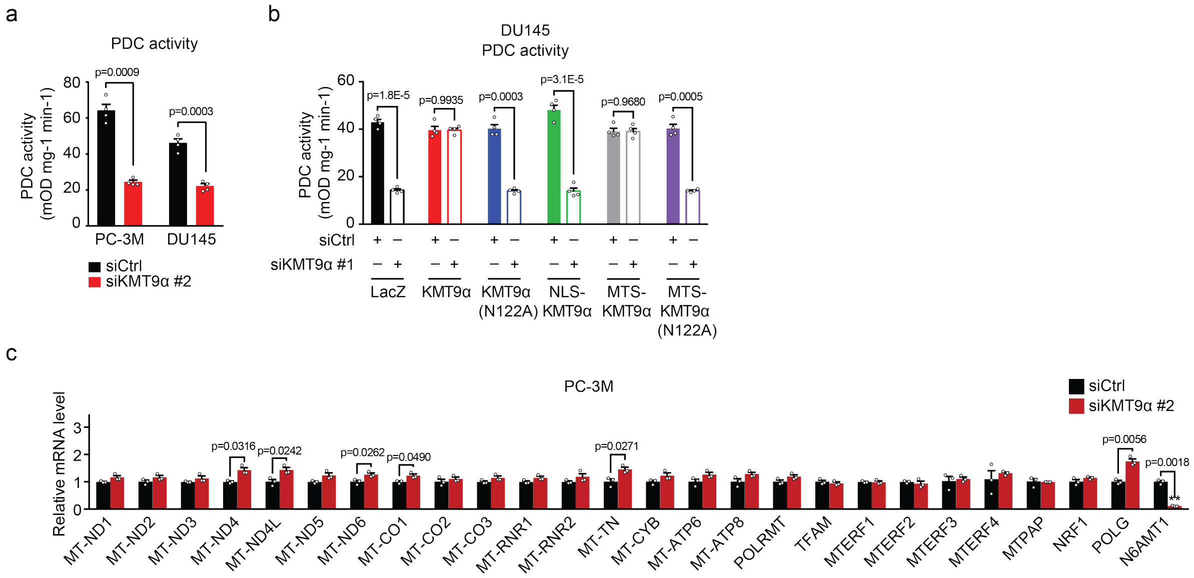

Supplementary Figure 2. Mitochondrial KMT9 regulates PDC activity in PCa cells. a, **b**, Activity of PDC immunocaptured from extracts of PC-3M or DU145 cells treated with siCtrl or siKMT9α (**a**) or with siCtrl or siKMT9α in combination with expression plasmid for LacZ, KMT9α, KMT9α (N122A), NLS-KMT9α, MTS-KMT9α, or MTS-KMT9α (N122A) as indicated (**b**). **c**, Relative mRNA levels determined by qRT-PCR of mitochondrial-encoded genes in PC-3M cells treated with siCtrl or siKMT9α. Data are presented as mean + SD (**a**, **b**: n=4 independent biological replicates per group; **c**: n=3 independent biological replicates per group). Statistical significance was determined by two-tailed Student's *t*-test. **a-c**, All experiments were independently repeated at least three times with similar results.

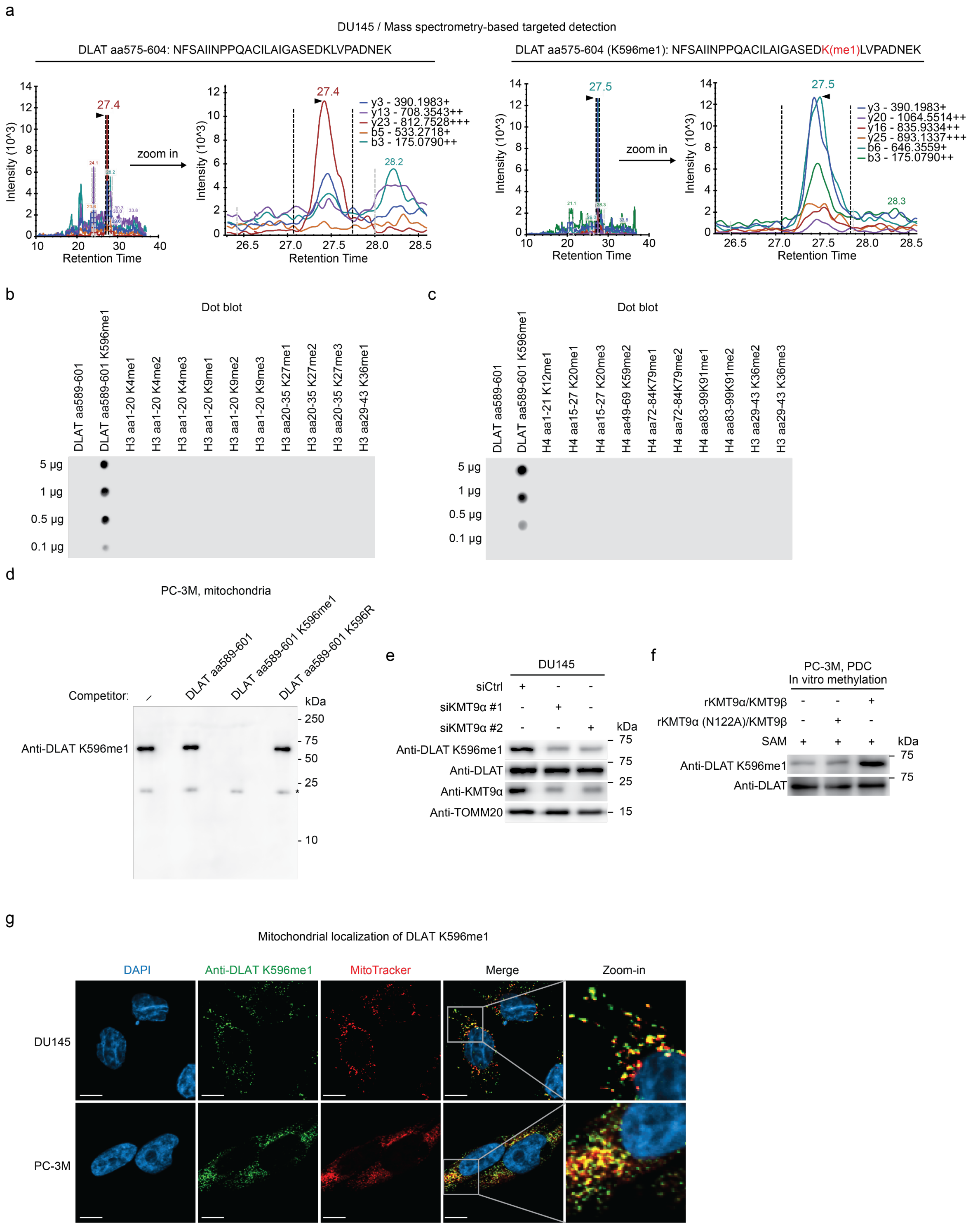

Supplementary Figure 3. Characterization of DLAT K596 methylation by KMT9. a, Targeted detection of DLAT methylation at K596 by mass spectrometry using mitochondrial extracts of DU145 cells. Unmethylated (left panel) and methylated (right panel) DLAT aa575-604 peptides were observed. The unmethylated peptide was detected at a retention time of 27.4 min together with fragmented signature ions y3, y13, y23, b5, and b3 (left panel). The corresponding peptide monomethylated at K596 from the same sample was detected at a

retention time of 27.5 min with the signature ions y3, y20, y16, y25, b6, and b3 (right panel). Mass-to-charge ratios and charges of signature ions are indicated. Of note, only the unmethylated ion y3 (602-NEK-604) was observed ruling out methylation at K604. **b**-**d**, Validation of anti-DLAT K596me1 antibody selectivity by dot blot (**b**, **c**) and Western blot (**d**). For dot blot, serial dilutions of the indicated peptides were blotted onto nitrocellulose membranes. Membranes were probed with anti-DLAT K596me1 antibody (**b**, **c**). Comparable results were obtained in two independent experiments. Western blot analysis was performed with mitochondrial extracts of PC-3M cells (**d**). Membranes were decorated with anti-DLAT K596me1 antibody in the absence or presence of the indicated DLAT peptides. **e**, Western blot analysis of mitochondrial fractions of DU145 cells treated with siCtrl or siKMT9α using anti-DLAT K596me1, anti-DLAT, or anti-KMT9α antibody as indicated. TOMM20 served as loading control. **f**, In vitro methylation of DLAT. Purified recombinant rKMT9α/β or rKMT9α (N122A)/KMT9β were incubated with SAM and PDC immunocaptured from PC-3M cells. Methylation reactions were analyzed by Western blot using anti-DLAT K596me1 or anti-DLAT antibody. **g**, Confocal fluorescence microscopy analysis of DLAT K596me1 distribution in PC-3M and DU145 cells using anti-DLAT K596me1 antibody. Mitochondria and nuclei were stained with MitoTracker and DAPI, respectively. Scale bar: 10 µm. **a**-**g**, All experiments were independently repeated at least three times with similar results.

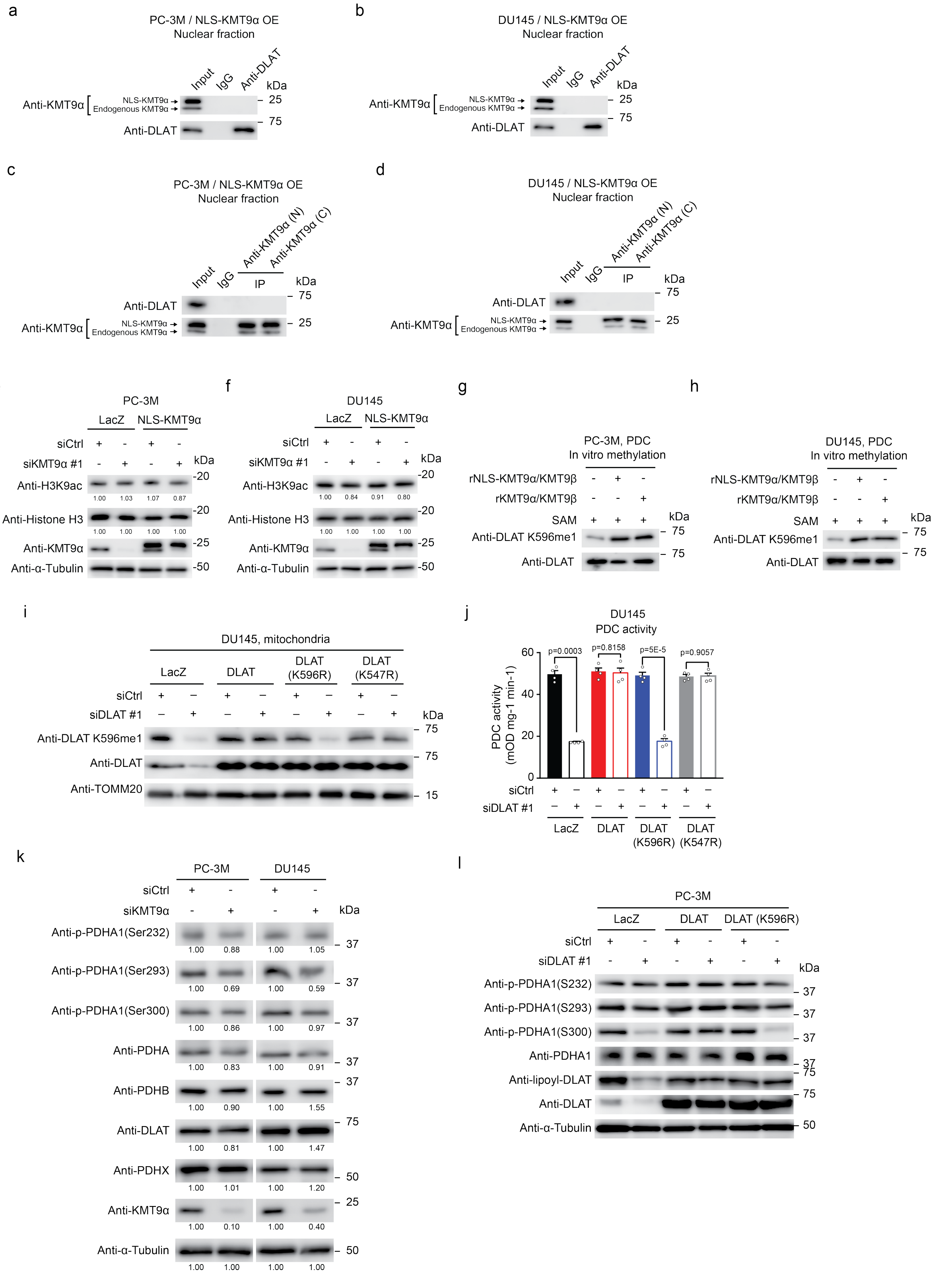

Supplementary Figure 4. Mitochondrial but not nuclear KMT9 regulates PDC activity through DLAT K596 methylation. a-d, Co-immunoprecipitation assays performed with anti-DLAT (**a**, **b**) or anti-KMT9α (**c**, **d**) antibodies and nuclear fractions of PC-3M (**a**, **c**) or DU145 (**b**, **d**) cells expressing NLS-KMT9α. Western blots were performed with the indicated

antibodies. **e**, **f**, Levels of H3K9ac present in PC-3M (**e** and DU145 cells (**f**) were analyzed by Western blot. Cells were transfected with siCtrl or siKMT9α in combination with expression plasmid for LacZ or NLS-KMT9α. Histone H3 and α-Tubulin served as controls. Numbers represent relative intensity values of the signals. **g**, **h**, In vitro methylation of DLAT. Purified recombinant rNLS-KMT9α/KMT9β was incubated with SAM and PDC immunocaptured from PC-3M (**g**) or DU145 (**h**) cells. Methylation reactions were analyzed by Western blot using anti-DLAT K596me1 or anti-DLAT antibody. **i**, Detection of DLAT K596me1 in mitochondrial extracts of DU145 cells transfected with siCtrl or siDLAT in combination with expression plasmid for LacZ, DLAT, DLAT (K596R), or DLAT (K547R) by Western blot. Membranes were probed with anti-DLAT K596me1 or anti-DLAT antibody. TOMM20 served as loading control. **j**, Activity of PDC immunocaptured from extracts of DU145 cells transfected with expression plasmid for LacZ, DLAT, DLAT (K596R), or DLAT (K547R) in combination with siCtrl or siDLAT as indicated. Data are presented as mean + SD (n=4 biological replicates). Statistical significance was determined by two-tailed Student's *t*-test. **k**, Western blot analysis to investigate abundance and phosphorylation of PDC components in PC-3M and DU145 cells transfected with siCtrl or siKMT9α. Membranes were decorated with the indicated antibodies. Numbers represent relative intensity values of the signals. The left band of the "band pairs" was set to 1.0. **l**, Western blot analysis to investigate abundance, phosphorylation, and lipoylation of PDC components in PC-3M cells transfected with siCtrl or siDLAT in combination with expression plasmid for LacZ, DLAT, or DLAT (K596R). Membranes were decorated with the indicated antibodies. **a**-**l**, All experiments were independently repeated at least three times with similar results.

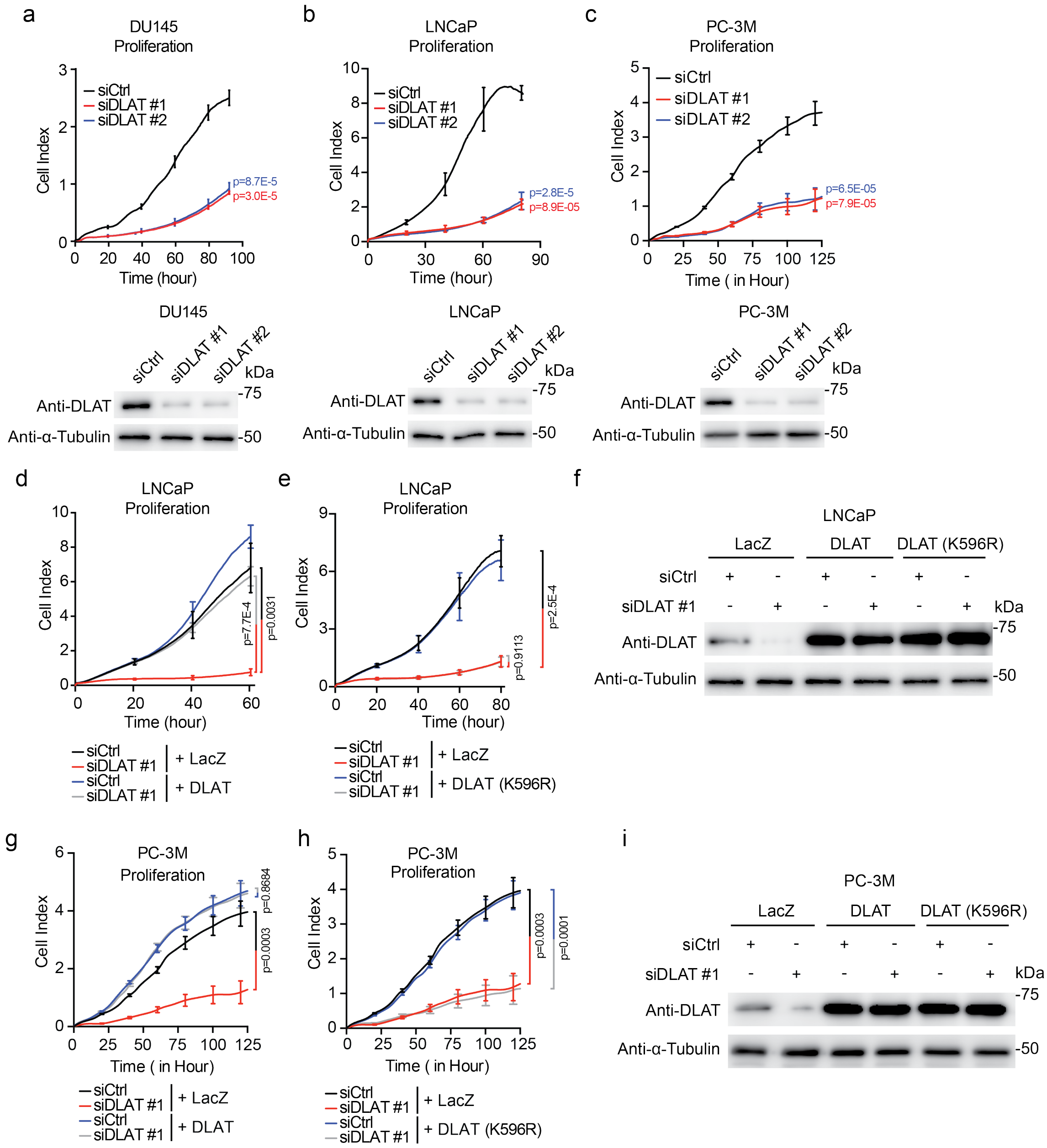

Supplementary Figure 5. DLAT K596 methylation is required for PCa cell proliferation. **a-c**, Proliferation of DU145 (**a**, upper panel), LNCaP (**b**, upper panel), and PC-3M (**c**, upper panel) cells transfected with siCtrl or siDLAT as indicated. DLAT knockdown efficiency was validated by Western blot (**a-c**, lower panels). α-Tubulin served as loading control. **d-i**, Proliferation of LNCaP (**d-f**) and PC-3M (**g-i**) cells transfected with siCtrl or siDLAT and expression plasmid for LacZ (**d-i**), DLAT (**d**, **f**, **g**, **i**) or DLAT (K596R) (**e**, **f**, **h**, **i**) as indicated. Knockdown efficiency and expression of exogenous DLAT proteins were validated by Western blot (**f**, **i**). α-Tubulin served as loading control. Data are presented as mean ± SD (**a-e**, **g**, **h**, n=4 biological replicates). Statistical significance was determined by two-tailed Student's *t*-test. **a-I**, All experiments were independently repeated at least three times with similar results.

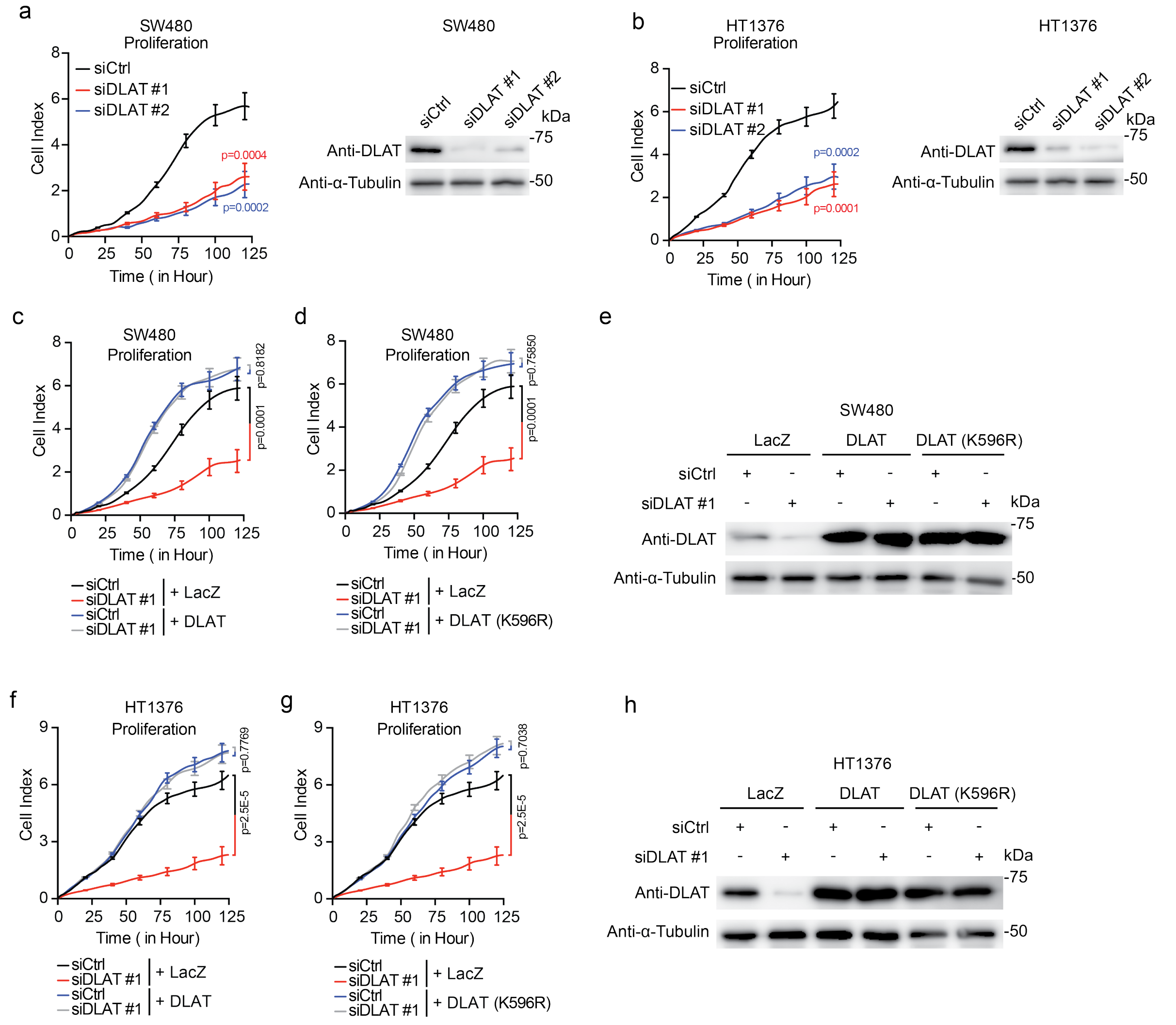

Supplementary Figure 6. DLAT K596 methylation is dispensable for non-PCa cell proliferation. a, **b**, Proliferation of SW480 (**a**, left panel), and HT1376 (**b**, left panel) cells transfected with siCtrl or siDLAT as indicated. DLAT knockdown efficiency was validated by Western blot (**a**, **b**, right panels). α-Tubulin served as loading control. **c-h**, Proliferation of SW480 (**c-e**), and HT1376 (**f-h**) cells transfected with siCtrl or siDLAT and expression plasmid for LacZ (**c-h**), DLAT (**c, e, f, h**) or DLAT (K596R) (**d**, **e**, **g**, **h**) as indicated. Knockdown efficiency and expression of exogenous DLAT proteins were validated by Western blot (**e**, **h**). α-Tubulin served as loading control. Data are presented as mean ± SD (**a-d**, **f**, **g**, n=4 biological replicates). Statistical significance was determined by two-tailed Student's *t*-test. **a-h**, All experiments were independently repeated at least three times with similar results.

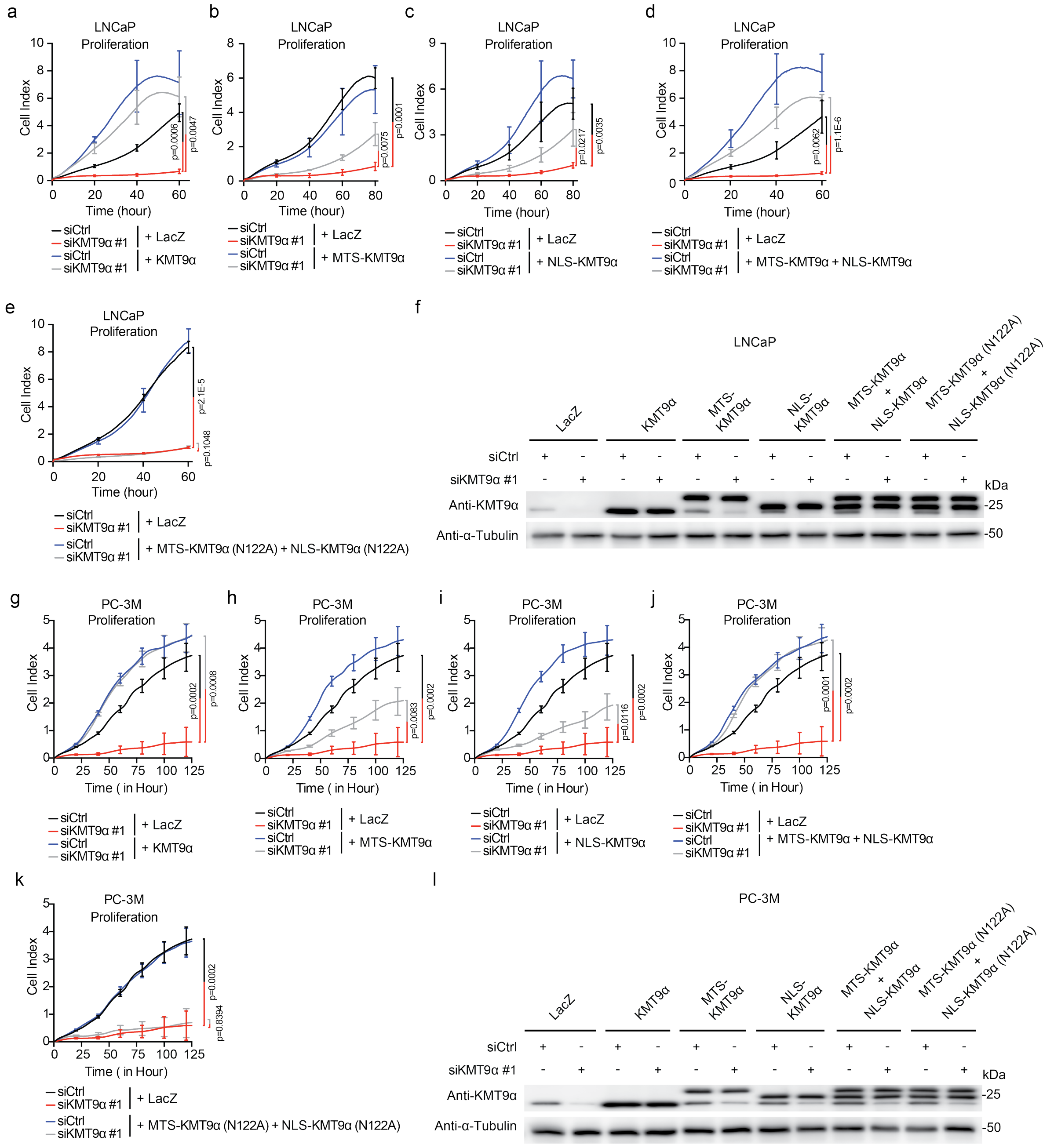

Supplementary Figure 7. Mitochondrial KMT9 is required for PCa cell proliferation. a-l, Proliferation of LNCaP (**a-f**) and PC-3M (**g-l**) cells transfected with siCtrl or siKMT9α and expression plasmid for LacZ (**a-l**), KMT9α (**a**, **g**), MTS-KMT9α (**b**, **d**, **h**, **j**), NLS-KMT9α (**c**, **d**, **i**, **j**), or MTS-KMT9α (N122A) and NLS-KMT9α (N122A) (**e**, **k**). Knockdown efficiency and expression of exogenous KMT9α proteins were validated by Western blot (**f**, **l**). α-Tubulin served as loading control. Data are presented as mean ± SD (**a-e**, **g-k**, n=4 biological replicates). Statistical significance was determined by two-tailed Student's *t*-test. **a-l**, All experiments were independently repeated at least three times with similar results.

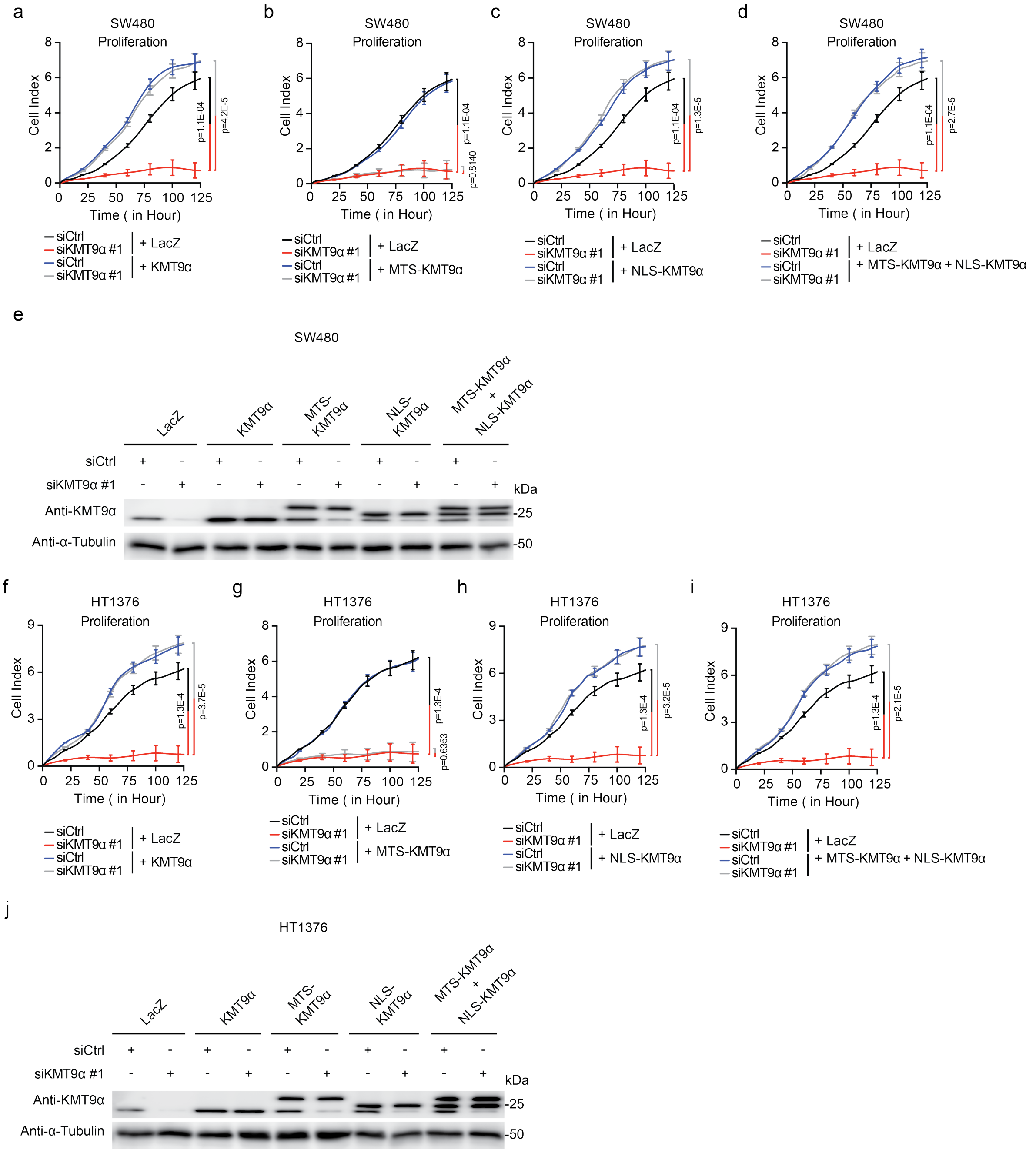

Supplementary Figure 8. Mitochondrial KMT9 is dispensable for non-PCa cell proliferation. a-j, Proliferation of SW480 (**a-e**) and HT1376 (**f-j**) cells transfected with siCtrl or siKMT9α and expression plasmid for LacZ (**a-j**), KMT9α (**a**, **f**), MTS-KMT9α (**b**, **d**, **g**, **i**), or NLS-KMT9α (**c**, **d**, **h**, **i**). Knockdown efficiency and expression of exogenous KMT9α proteins were validated by Western blot (**e**, **j**). α-Tubulin served as loading control. Data are presented as mean ± SD (**a-d**, **f-i**, n=4 biological replicates). Statistical significance was determined by two-tailed Student's *t*-test. **a-j**, All experiments were independently repeated at least three times with similar results.

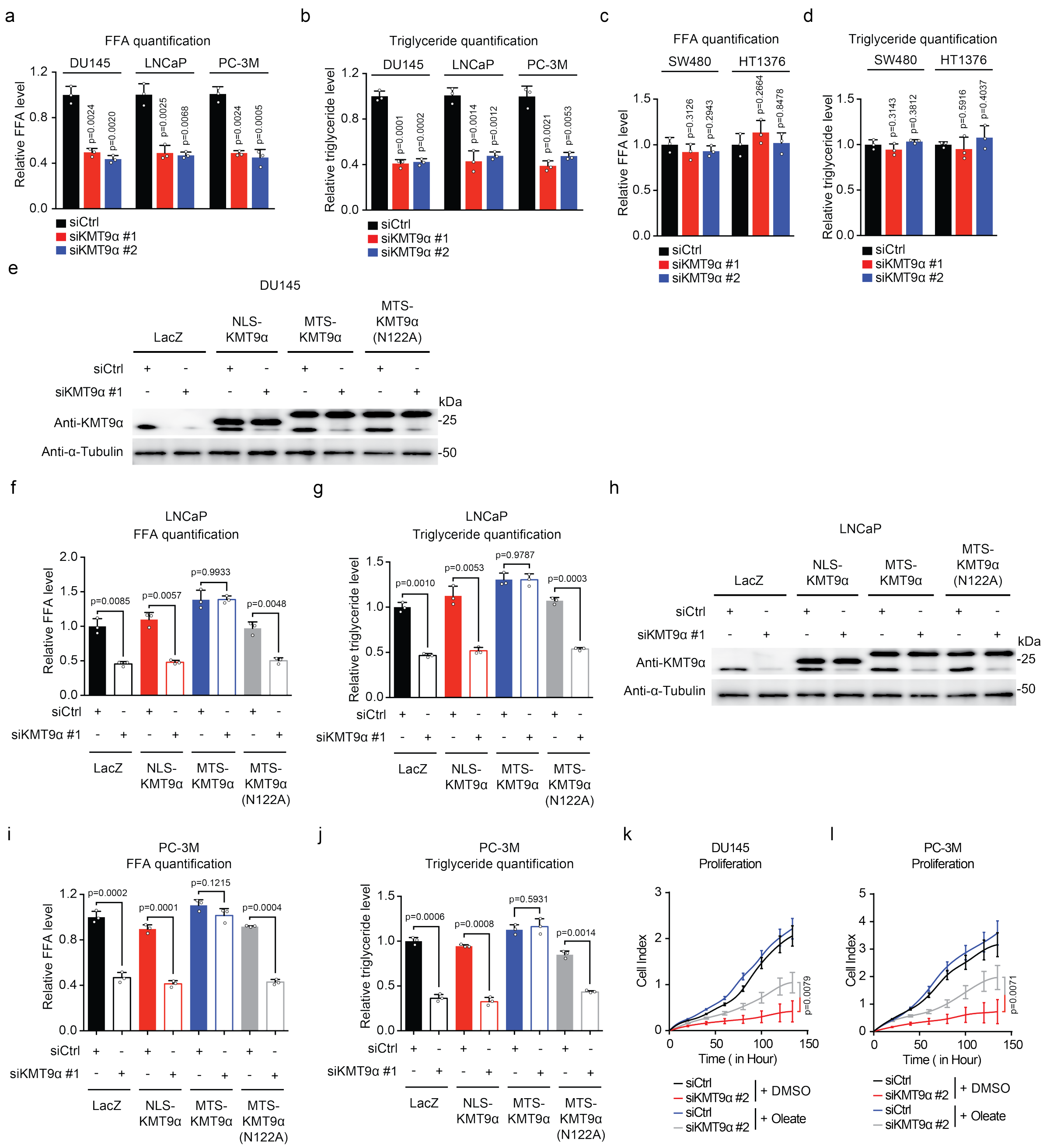

Supplementary Figure 9. Mitochondrial KMT9 regulates PCa cell proliferation via de novo lipogenesis. an-aq, Free fatty acid (**a**, **c**) and triglyceride (**b**, **d**) levels in DU145 (**a**, **b**), LNCaP (**a**, **b**), PC-3M (**a**, **b**), SW480 (**c**, **d**), and HT1376 (**c**, **d**) cells transfected with siCtrl or siKMT9α as indicated. **e**, Validation of knockdown efficiency and expression of exogenous KMT9α proteins in DU145 cell by Western blot (control for Fig. 3k, l). **f-j**, Free fatty acid (**f**, **i**) and triglyceride (**g**, **j**) levels in LNCaP and PC-3M cells transfected with siCtrl or siKMT9α in combination with expression plasmid for LacZ, NLS-KMT9α, MTS-KMT9α, or MTS-KMT9α (N122A). Knockdown efficiency and expression of exogenous KMT9α proteins was validated by Western blot (**h**). α-Tubulin served as loading control. **k**, **l**, Proliferation of DU145 (**k**) and PC-3M (**l**) cells transfected with siCtrl or siKMT9α in the presence or absence of oleate (25

μM) to detect potential effects of co-treatment. Data are presented as mean + SD (**a**-**d**, **f**, **g**, **i**, **j**, n=3 biological replicates) or mean ± SD (**k**, **l**, n=4 biological replicates). Statistical significance was determined by two-tailed Student's *t*-test. **a**-**l**, All experiments were independently repeated at least three times with similar results.

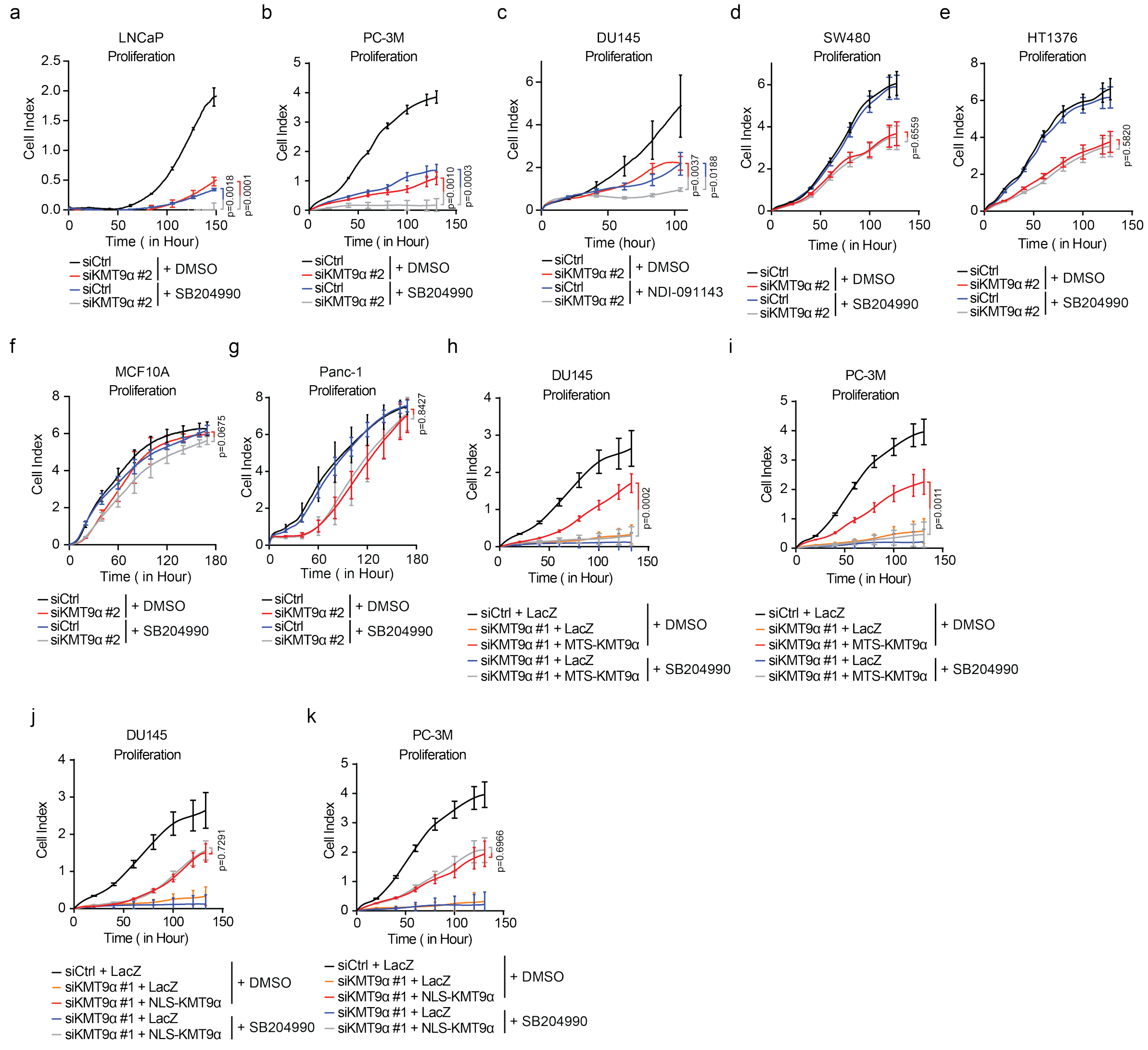

Supplementary Figure 10. Combined targeting of de novo lipogenesis and KMT9 efficiently suppresses PCa cell growth at suboptimal doses. a, **b**, Proliferation of LNCaP (**a**) and PC-3M (**b**) cells transfected with sub-optimal concentrations of siCtrl or siKMT9α in the presence or absence of 100 μM SB204990 to detect potential effects of co-treatment. **c**, Proliferation of DU145 cells transfected with sub-optimal concentrations of siCtrl or siKMT9α in the presence or absence of 60 μM NDI-091143 to detect potential effects of co-treatment. **d-g**, Proliferation of SW480 (**d**), HT1376 (**e**), MCF10A (**f**), Panc-1 (**g**) cells transfected with sub-optimal concentrations of siCtrl or siKMT9α in the presence or absence of 100 μM SB204990 to detect potential effects of co-treatment. **h-k**, Proliferation of DU145 (**h**, **j**) and PC-3M (**i**, **k**) cells transfected with siCtrl or siKMT9α in combination with LacZ, MTS-KMT9α or NLS-KMT9α expression plasmid, followed by treatment with DMSO or 100 μM SB204990. Data are presented as mean ± SD (**a-k**, n=4 biological replicates). Statistical significance was determined by two-tailed Student's *t*-test. **a-k**, All experiments were independently repeated at least three times with similar results.

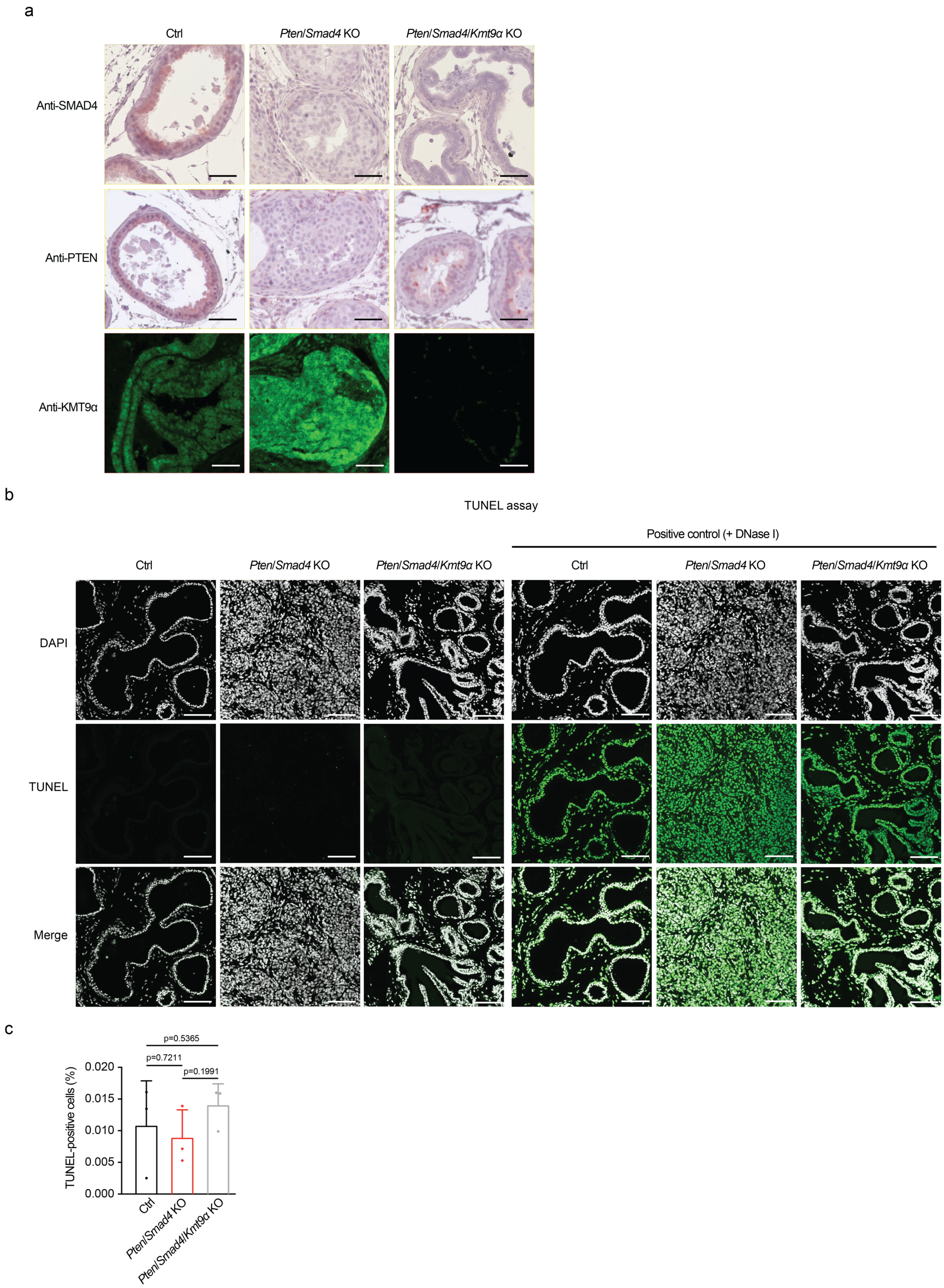

Supplementary Figure 11. Loss of KMT9 does not affect cell death in *Pten*/*Smad4* KO mice. a, Expression of SMAD4, PTEN, and KMT9α in prostates of Ctrl, *Pten*/*Smad4* KO, and *Pten*/*Smad4*/*Kmt9α* KO mice. Tissue sections were stained by immunohistochemistry

(SMAD4, PTEN) or with Akoya Opal dye (KMT9α). Scale bars: 50 µm. **b**, **c**, Representative fluorescence microscopy images showing TUNEL staining in control (Ctrl), *Pten*/*Smad4* KO, and *Pten*/*Smad4/Kmt9α* KO prostates. Nuclei were stained with DAPI (upper panels, gray). DNase I-treated prostate sections served as positive controls (right panels). Scale bars: 100 µm. The corresponding quantification of TUNEL-positive cells is shown in **c**. **c**, n=3 microscopic fields per group. Data are presented as mean + SD. Statistical significance was determined by two-tailed Student's *t*-test. **a**-**c**, All experiments were independently repeated at least three times with similar results.

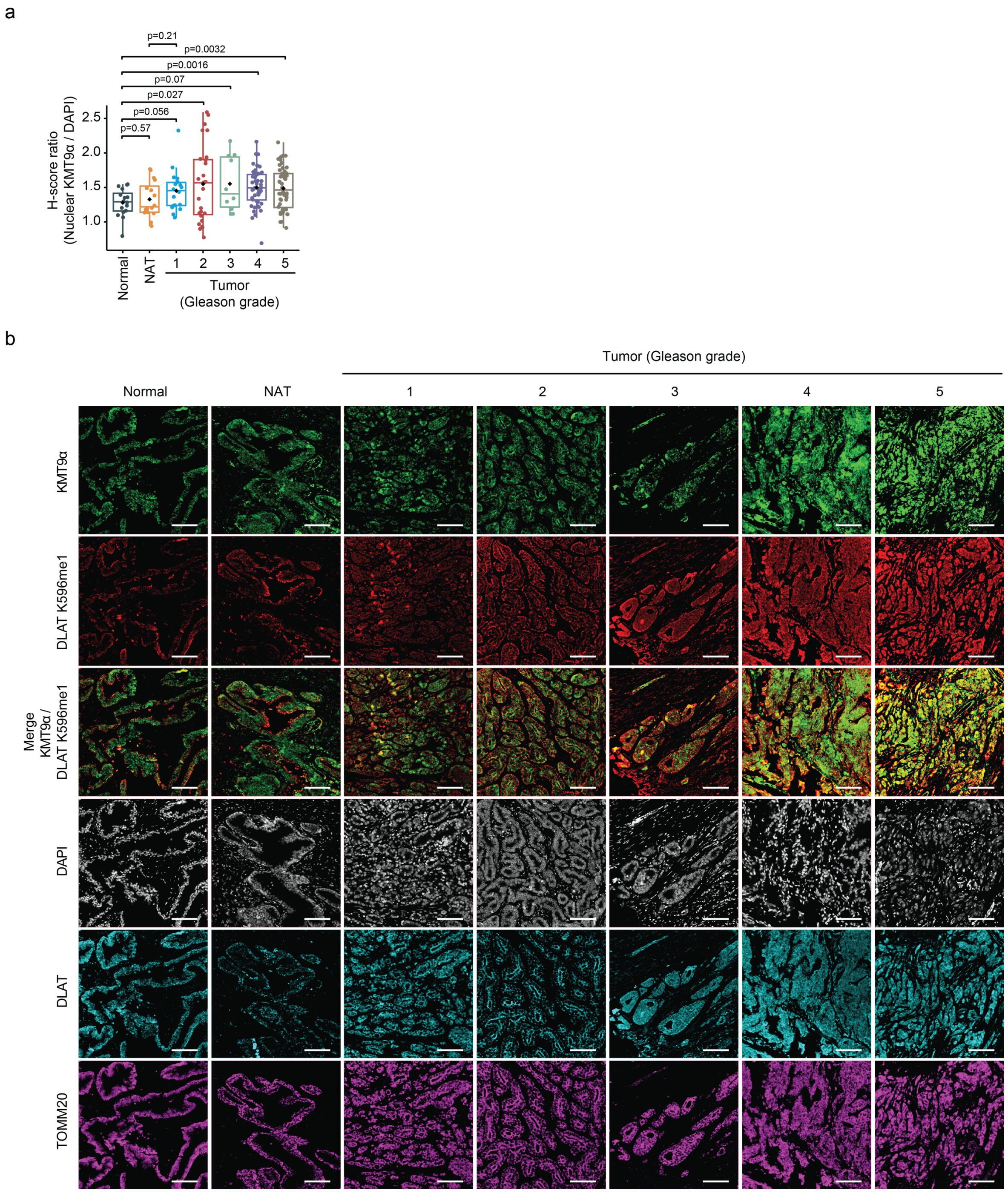

Supplementary Figure 12. Nuclear KMT9α level is elevated in high Gleason grade PCa patient specimens. a, H-score ratios (normalized signal intensity ratios) for nuclear KMT9α normalized to DAPI in normal (n=16 tissue microarray cores), normal adjacent tissue (NAT, n=16 cores), and PCa tissues of Gleason grades 1-5 (n=18,27,10,48,52 cores respectively) based on immunofluorescence images. Statistical significance was determined by two-tailed Student's *t*-test. **b**, Multiplexed immunofluorescence analysis of KMT9α, DLAT, and TOMM20 expression as well as DLAT K596me1 levels in tissue sections representing normal, normal adjacent, and PCa tissue with different Gleason grades obtained from human patients. Tissue

sections were stained with DAPI and Akoya Opal dye. Representative images are shown. Scale bars: 100 μm. **a**, **b**, All experiments were independently repeated at least three times with similar results.
