# Supplementary material for: Mitochondrial KMT9 methylates DLAT to control pyruvate dehydrogenase activity and prostate cancer growth
Source: Nat Commun. 2025 Jan 30;16:1191. doi: 10.1038/s41467-025-56492-8 (PMC11782658; doi:10.1038/s41467-025-56492-8)
Supplement: Supplementary file 2 — Description of Additional Supplementary Files [file 41467_2025_56492_MOESM2_ESM.pdf]

Description of Additional Supplementary Files

Supplementary Data 1: List of all proteins co-immunoprecipitated with KMT9a using two different antibodies targeting the C-terminus (27630, C) and N-terminus (28445, N) of KMT9a, followed by LC-MS/MS analysis.

Supplementary Data 2: List of significantly altered metabolites identified by UPLC-MS/MS-based metabolomics analysis in PC-3M cells after 3-day treatment with siKMT9a compared to siCtrl.

Supplementary Data 3: Information for plasmids, antibodies, and siRNAs used in this study.
