# Supplementary material for: Mitochondrial KMT9 methylates DLAT to control pyruvate dehydrogenase activity and prostate cancer growth
Source: Nat Commun. 2025 Jan 30;16:1191. doi: 10.1038/s41467-025-56492-8 (PMC11782658; doi:10.1038/s41467-025-56492-8)
Supplement: Supplementary file 6 — Reporting Summary [file 41467_2025_56492_MOESM6_ESM.pdf]

nature portfolio

Corresponding author(s): Prof. Roland Schüle
Dr. Eric Metzger

Last updated by author(s): Dec 6, 2024

Reporting Summary

Nature Portfolio wishes to improve the reproducibility of the work that we publish. This form provides structure for consistency and transparency in reporting. For further information on Nature Portfolio policies, see our Editorial Policies and the Editorial Policy Checklist.

Statistics

For all statistical analyses, confirm that the following items are present in the figure legend, table legend, main text, or Methods section.

n/a	Confirmed	
☐	☒	The exact sample size (*n*) for each experimental group/condition, given as a discrete number and unit of measurement
☐	☒	A statement on whether measurements were taken from distinct samples or whether the same sample was measured repeatedly
☐	☒	The statistical test(s) used AND whether they are one- or two-sided<br>*Only common tests should be described solely by name; describe more complex techniques in the Methods section.*
☒	☐	A description of all covariates tested
☐	☒	A description of any assumptions or corrections, such as tests of normality and adjustment for multiple comparisons
☐	☒	A full description of the statistical parameters including central tendency (e.g. means) or other basic estimates (e.g. regression coefficient) AND variation (e.g. standard deviation) or associated estimates of uncertainty (e.g. confidence intervals)
☐	☒	For null hypothesis testing, the test statistic (e.g. *F*, *t*, *r*) with confidence intervals, effect sizes, degrees of freedom and *P* value noted<br>*Give P values as exact values whenever suitable.*
☒	☐	For Bayesian analysis, information on the choice of priors and Markov chain Monte Carlo settings
☒	☐	For hierarchical and complex designs, identification of the appropriate level for tests and full reporting of outcomes
☐	☒	Estimates of effect sizes (e.g. Cohen's *d*, Pearson's *r*), indicating how they were calculated

Our web collection on statistics for biologists contains articles on many of the points above.

Software and code

Policy information about availability of computer code

Data collection	*Provide a description of all commercial, open source and custom code used to collect the data in this study, specifying the version used OR state that no software was used.*
Data analysis	Statistics: Excel (Microsoft 365), GraphPad Prism (version 7.00), QuPath(version 0.5.0), MaxQuant (version 1.6.6.0 53), Perseus (version 2.1.3.0)

For manuscripts utilizing custom algorithms or software that are central to the research but not yet described in published literature, software must be made available to editors and reviewers. We strongly encourage code deposition in a community repository (e.g. GitHub). See the Nature Portfolio guidelines for submitting code & software for further information.

Data

Policy information about availability of data

All manuscripts must include a data availability statement. This statement should provide the following information, where applicable:

- Accession codes, unique identifiers, or web links for publicly available datasets
- A description of any restrictions on data availability
- For clinical datasets or third party data, please ensure that the statement adheres to our policy

The targeted mass spectrometry data for DLAT methylation detection presented in supplementary Fig. 3a have been deposited in the PeptideAtlas SRM Experiment

Library (PASSEL) via ProteomeXchange under accession code PASS05878 (http://www.peptideatlas.org/PASS/PASS05878). The LC-MS/MS data for KMT9a interactome analysis presented in Fig. 1f have been deposited to the ProteomeXchange Consortium via the PRIDE partner repository under accession code PXD053942 (https://www.ebi.ac.uk/pride/archive/projects/PXD053942). The metabolomics data in Fig. 1h-l have been deposited to the MetaboLights database with the dataset identifier MTBLS2452 (https://www.ebi.ac.uk/metabolights/MTBLS2452). Source data are provided with this paper.

Human research participants

Policy information about studies involving human research participants and Sex and Gender in Research.

Reporting on sex and gender	This study utilized tissue specimens exclusively from male donors (n=10). The biological sex of donors was determined from medical records. Male donors were specifically selected for all tissue types (including non-prostate tissues) to eliminate potential sex-based variations in our control analyses
Population characteristics	Our study included tissue specimens from 10 male donors, including: 4 normal prostate tissues from individuals without prostate malignancy, 3 prostate cancer tissues obtained by transurethral resection from patients with histologically confirmed prostate cancer, 1 normal colon tissue obtained from regions proximal to colorectal adenocarcinoma lesions, 1 bladder cancer tissue obtained by transurethral resection, 1 normal mesenchymal tissue obtained from mesocolonic tissue. The non-neoplastic colon and mesenchymal tissues were collected during laparoscopic colon surgery and evaluated macroscopically in situ. While these control tissues were obtained from subjects with colorectal adenocarcinoma diagnosis, they were specifically collected from regions proximal to the tumor and confirmed to be free of malignancy through macroscopic assessment.
Recruitment	Tissue specimens were obtained through different surgical procedures: laparoscopic colon surgery for colon and mesenchymal tissues, and transurethral resection for bladder and prostate specimens. Bladder cancer tissue was identified through macroscopic assessment, while prostate cancer samples were collected from patients with pre-existing cancer diagnosis.
Ethics oversight	The study was conducted under two ethical approvals from the Ethics Committee of the University of Freiburg:<br><br>Collection of colon and mesenchymal tissues: ETK: 21-1162_5<br>Collection of bladder and prostate specimens: ETK: 266/14

Note that full information on the approval of the study protocol must also be provided in the manuscript.

Field-specific reporting

Please select the one below that is the best fit for your research. If you are not sure, read the appropriate sections before making your selection.

☒ Life sciences ☐ Behavioural & social sciences ☐ Ecological, evolutionary & environmental sciences

For a reference copy of the document with all sections, see nature.com/documents/nr-reporting-summary-flat.pdf

Life sciences study design

All studies must disclose on these points even when the disclosure is negative.

Sample size	Sample sizes were determined based on previous similar experiments in the field to ensure adequate statistical power. These sample sizes have been demonstrated to reliably detect biologically meaningful differences between experimental groups.
Data exclusions	We did not exclude data
Replication	To verify the reproducibility of the experimental findings, we repeated the experiments. Experiments were done the first time by one given investigator and then repeated by another investigators. All experiments were independently repeated at least three times with similar results. All attempts at replication were successful.
Randomization	No randomization have been used in this study
Blinding	Investigators were not blinded during experiments analysis and outcome assessment. In this study, blinding was not implemented because all data were collected and analyzed using objective, automated measurements and standardized computational methods, which minimized potential subjective bias. The quantitative nature of our experimental readouts and analysis pipeline did not require investigator blinding to ensure unbiased assessment of results.

Reporting for specific materials, systems and methods

We require information from authors about some types of materials, experimental systems and methods used in many studies. Here, indicate whether each material, system or method listed is relevant to your study. If you are not sure if a list item applies to your research, read the appropriate section before selecting a response.

Materials & experimental systems

n/a	Involved in the study
	☒ Antibodies
	☒ Eukaryotic cell lines
☒	☐ Palaeontology and archaeology
	☒ Animals and other organisms
☒	☐ Clinical data
☒	☐ Dual use research of concern

Methods

n/a	Involved in the study
☒	☐ ChIP-seq
☒	☐ Flow cytometry
☒	☐ MRI-based neuroimaging

Antibodies

Antibodies used

Anti-KMT9a (#28445, Schüle Lab, dilutions: 1:400 (WB), 1:100 (IF and Akoya opal dye staining), 5μg per 1mg lysate (IP)),
Anti-KMT9a (#27630, Schüle Lab, dilutions: 5μg per 1mg lysate (IP)),
Anti-KMT9b (#28358, Schüle Lab, dilutions: 1:500 (WB)),
Anti-α-Tubulin (T6074, Sigma, dilutions: 1:5000 (WB)),
Anti-Lamin A (ab26300, Abcam, dilutions: 1:500 (WB)),
Anti-Tom20 (sc-11415, Santa Cruz, dilutions: 1:500 (WB), 1:100 (Akoya opal dye staining)),
Anti-TFAM (#7495, Cell Signaling Technology, dilutions: 1:1000 (WB)),
Anti-Cytochrome C (CYCS) (TA326782, OriGene, dilutions: 1:500 (WB)),
Anti-DLAT K596me1 (#31453, Schüle Lab, dilutions: 1:1000 (WB), 1:100 (Akoya opal dye staining)),
Anti-H3K9ac (pAb-ACHAHS-044, Diagenode, dilutions: 1:1000 (WB)),
Anti-Histone H3 (#4499, Cell Signaling Technology, dilutions: 1:2000 (WB)),
Anti-PDH-E1α (PDHA1 phospho S232) (AP1063, Sigma, dilutions: 1:1000 (WB)),
Anti-PDHA1 (phospho S293) (ab92696, Abcam, dilutions: 1:1000 (WB)),
Anti-PDH-E1α (PDHA1 phospho S300) (AP1064, Sigma, dilutions: 1:1000 (WB)),
Anti-PDHA (#2784, Cell Signaling Technology, dilutions: 1:1000 (WB)),
Anti-PDHB (14744-1-AP, Proteintech, dilutions: 1:1000 (WB)),
Anti-DLAT (#12362S, Cell Signaling Technology, dilutions: 1:1000 (WB), 1:100 (Akoya opal dye staining), 10μl per 1mg lysate (IP)),
Anti-PDHX (10951-1-AP, Proteintech, dilutions: 1:2000 (WB)),
Anti-pan methyl lysine (ab7315, Abcam, dilutions: 1:1000 (WB)),
Anti-Lipoic Acid (#437695, Merck Millipore, dilutions: 1:1000 (WB)),
Anti-PTEN (#9559, Cell Signaling Technology, dilutions: 1:200 (IHC)),
Anti-SMAD4 (#46535, Cell Signaling Technology, dilutions: 1:400 (IHC)),
Anti-Ki67 (PN 232179, Akoya Biosciences, dilutions: 1:200 (Akoya staining)),
Anti-PCNA (4550124, Akoya Biosciences, dilutions: 1:400 (Akoya staining))

Validation

The validation of anti-KMT9a and anti-KMT9b are described in (E Metzger, 2019). The validation of anti-DLAT K596me1 is described in supplementary figure 3b-f of the manuscript. The other antibodies were sourced from commercial suppliers. Additional details for each antibody are available from the supplier's websites.

Eukaryotic cell lines

Policy information about cell lines and Sex and Gender in Research

Cell line source(s)

All cell lines used in this study were obtained from commercial sources: DU145 (American Type Culture Collection, cat. no.: HTB-81), PC-3M (Tebu-Bio, cat. no.: 305061), LNCaP (German Collection of Microorganisms and Cell Cultures, cat. no.: ACC 256), C4-2B (Thermo Fisher Scientific, cat. no.: 50-238-4833), HepG2 (American Type Culture Collection, cat. no.: HB-8065), SW480 (European Collection of Cell Cultures, cat. no.: 87092801), HT1376 (Tebu-Bio, cat. no.: 305100), MCF10A (Merck, cat. no.: CLLS1069), and Panc-1 (Thermo Fisher Scientific, cat. no.: 50-238-2594).

Authentication

None of the cell line used has been authenticated

Mycoplasma contamination

We confirm that all cell line were tested negative for mycloplasma contamination

Commonly misidentified lines
(See ICLAC register)

No cell line used is listed in the database of commonly missidentified cell lines

Animals and other research organisms

Policy information about studies involving animals; ARRIVE guidelines recommended for reporting animal research, and Sex and Gender in Research

Laboratory animals

Nkx3.1-Cre-ERT2(Tg/Tg)/Ptenfl/fl/Smad4fl/fl, Nkx3.1-Cre-ERT2(Tg/Tg)/Ptenfl/fl/Smad4fl/fl/Kmt9afl/fl mice were used for in vivo

	experiments. By treatment of ten weeks old mice with tamoxifen for eight weeks, we obtained prostate-specific Pten/Smad4 KO and Pten/Smad4/Kmt9a KO mice.
Wild animals	The study did not involve wild animals.
Reporting on sex	This study investigated prostate cancer, which only occurs in males. The prostate gland is a part of the male reproductive system, and females do not have a prostate. Therefore, the study design only included male subjects, as the research question was sex-specific. All animals used in the experiments were male to appropriately model the disease under investigation
Field-collected samples	Mice were maintained under temperature- and humidity-controlled conditions with a 12-h light/dark cycle, free access to water, and a standard (3807, granovit, Kaiseraugust, Switzerland) or 400 mg/kg tamoxifen citrate-containing (A115T70400, ssniff, Soest, Germany) rodent chow.
Ethics oversight	All mice were housed in the pathogen-free barrier facility of the University Medical Center Freiburg in accordance with institutional guidelines and approved by the regional board (Regierungspräsidium Freiburg, 35-9185.81/G-23/011).

Note that full information on the approval of the study protocol must also be provided in the manuscript.
